# Optimal slice thickness for improved accuracy of quantitative analysis of fluorescent cell and microsphere distribution in cryo-images

**DOI:** 10.1038/s41598-023-37927-y

**Published:** 2023-07-05

**Authors:** Patiwet Wuttisarnwattana, Brendan L. Eck, Madhusudhana Gargesha, David L. Wilson

**Affiliations:** 1 Biomedical Engineering Institute, Department of Computer Engineering, Excellence Center in Infrastructure Technology and Transportation Engineering, Chiang Mai University, Chiang Mai, 50200 Thailand; 2 Imaging Institute, Cleveland Clinic, Cleveland, OH 44195 USA; 3 BioInVision Inc., Mayfield Village, OH 44143 USA; 4 Department of Biomedical Engineering, Case Western Reserve University, Cleveland, OH 44106 USA

**Keywords:** Biomedical engineering, Computer science, Biological fluorescence, Cellular imaging, Fluorescence imaging, Molecular imaging, Optical imaging, Wide-field fluorescence microscopy, Blood flow, Mesenchymal stem cells

## Abstract

Cryo-imaging has been effectively used to study the biodistribution of fluorescent cells or microspheres in animal models. Sequential slice-by-slice fluorescent imaging enables detection of fluorescent cells or microspheres for corresponding quantification of their distribution in tissue. However, if slices are too thin, there will be data overload and excessive scan times. If slices are too thick, then cells can be missed. In this study, we developed a model for detection of fluorescent cells or microspheres to aid optimal slice thickness determination. Key factors include: section thickness (*X*), fluorescent cell intensity (*I*_*fluo*_), effective tissue attenuation coefficient (*μ*_*T*_), and a detection threshold (*T*). The model suggests an optimal slice thickness value that provides near-ideal sensitivity while minimizing scan time. The model also suggests a correction method to compensate for missed cells in the case that image data were acquired with overly large slice thickness. This approach allows cryo-imaging operators to use larger slice thickness to expedite the scan time without significant loss of cell count. We validated the model using real data from two independent studies: fluorescent microspheres in a pig heart and fluorescently labeled stem cells in a mouse model. Results show that slice thickness and detection sensitivity relationships from simulations and real data were well-matched with 99% correlation and 2% root-mean-square (RMS) error. We also discussed the detection characteristics in situations where key assumptions of the model were not met such as fluorescence intensity variation and spatial distribution. Finally, we show that with proper settings, cryo-imaging can provide accurate quantification of the fluorescent cell biodistribution with remarkably high recovery ratios (number of detections/delivery). As cryo-imaging technology has been used in many biological applications, our optimal slice thickness determination and data correction methods can play a crucial role in further advancing its usability and reliability.

## Introduction

Cryo-imaging is a 3D microscopic imaging technology that enables localization of fluorescent cells everywhere in a whole mouse or rat with single cell sensitivity^1–9^. The system consists of a motorized microtome cryostat, a microscope with brightfield and fluorescent capacities, a robotic positioner, and control software. To perform cryo-imaging, the tissues of interest are flash-frozen using liquid nitrogen and fixed to a sectioning stage and alternating sectioning-and-imaging is employed. The system acquires tiled, large field of view, high resolution (~ 10 µm), color brightfield and fluorescent images of the tissue. By stacking image slices, 3D image volumes can be generated for biodistribution visualization and analysis. These exceptional features make cryo-imaging unique as compared to other 3D in vivo modalities such as magnetic resonance imaging (MRI) or bioluminescence imaging (BLI). While such in vivo imaging modalities can image a whole animal, they lack adequate resolution and can produce only grayscale images. With the aforementioned features, cryo-imaging addresses a critical gap in other biological research imaging modalities.

Cryo-imaging has been used to study biodistributions of fluorescent cells or microspheres in various animal models, including small rodents^2–4,6,9–20^, dogs^7,21^, pigs^5,22,23^, and other animal models^24^. Burden-Gulley et al.^13–15^ used cryo-imaging to visualize migratory and invasive behaviors of glioblastoma cells in a mouse model. Recently, we developed a cryo-imaging based platform to quantify and evaluate fluorescent metastases throughout the whole mouse body^4,17–19^. The platform was found to be suitable for the evaluation and optimization of pipelines of technologies (imaging agents, imaging methods, therapeutics, tumor models, etc.) which are essential for detecting, understanding, and treating metastatic cancer. Many groups^7,8,24,25^ including our own^5,26^, employed the technology to spatially resolve quantitative, high-resolution 3D myocardial perfusion via the fluorescent microsphere entrapment method. Van Horssen at el. also used the technology to visualize biodistribution of both fluorescent microspheres^7,21–23,27^ and fluorescently labeled monocytes^6^ to investigate properties of coronary neovascularization progression in animal hearts. Moreover, we have previously used cryo-imaging technology to study a whole body biodistribution of intravenously injected stem cells and disease-inducing immune cells in a graft-versus-host disease (GVHD) mouse model^2,3,9–12,20,28,29^. Examples of the fluorescent images showing the microsphere signals and the stem cell signals are shown in the Supplemental Document. Given the high utility of cryo-imaging in biomedical imaging research, there have been increasingly numerous applications utilizing this technology.

Although cryo-imaging has been widely used, the optimal value of the slice thickness has not been identified before. In some experiments that require imaging of a thick tissue (e.g., pig heart), a large value of section thickness (e.g., X > 100 µm) is often chosen. The benefits of a larger section thickness include faster imaging and computing times, less memory usage, and longer life for the sectioning knife. However, signals of dimly fluorescent cells embedded in a thick tissue may not reach the surface and may not be detected by the imaging system. The physics of signal loss can be described by the principles of light absorption and scattering in the biological tissue^30–33^. In general, by setting a slice thickness that is too large, a significant rate of false negatives can be expected. This would render the results unreliable, particularly in applications that demand accurate absolute quantification. On the other hand, if one sets the slice thickness to be too small, although accurate quantification can be obtained, it would not be computationally or economically efficient. For example, a tissue which can be imaged overnight (12 h) at 120 µm slice thickness will take 3 days (72 h) to scan at 20 µm. There is expected to be an optimal value for slice thickness that will give accurate cell/microsphere counting at a reasonable scan time.

In this study, we propose a model for estimating the optimal slice thickness, which is the thickest slice that maintains accurate cell counts. We validate the model on data and determine for the first time the relationship between slice thickness and detectability. We then compare the results between simulated data and real data to determine their degree of correlation.

## Theory

### The optimal slice thickness model

**Figure 1:**
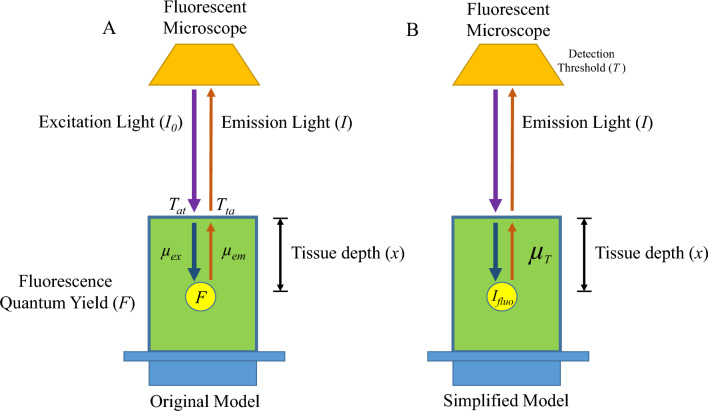
Epifluorescence imaging model. Figure (**A**) shows that a fluorescent labeled cell (yellow circle) is embedded in a homogeneous tissue (green box). Excitation/incident light from a fluorescent light source travels from a microscope above (orange trapezoid) and then interacts with the fluorophore of the cells. The emitted light travels back to the camera where the cell signal is determined to be detected or not by a detection threshold. The model can further be simplified to figure (**B**).

The model is developed principally based on a previous study on removal of out-of-plane fluorescent signals^34^. Fluorescent cells are assumed to be embedded in a homogeneous tissue volume (Fig. 1). By performing the cryo-imaging, the top thin layer of the volume is sectioned away. Images of the remaining block-face are then acquired via epifluorescence imaging, with the detected signal intensity determined according to optical properties of the tissue. The model aims to determine whether or not the imaging system can detect signals of the fluorescent cells below the exposed surface (block-face).

Let us assume that photons from a fluorescent light source, with an intensity *I*_*0*_, are incident on the specimen block-face (Fig. 1). At the air-specimen interface, a fraction of incident light is transmitted into the tissue, *T*_*at*_, dependent upon the block-face index of refraction. Excitation photons that enter the specimen are absorbed and scattered into the tissue with an effective tissue attenuation coefficient *μ*_*ex*_ (cm^−1^). Transmitted photons continue through the tissue until incident on a fluorophore at depth *x* below the surface. A fraction, *F*, of these incident photons is absorbed by the fluorophore and results in fluorescent emission of photons at a lower energy (Stokes shift) in the direction of the imaging system. The emitted photons are scattered and absorbed within the tissue with another effective tissue attenuation coefficient *μ*_*em*_ (cm^−1^), and a percentage are transmitted at the tissue-air interface, *T*_*ta*_. The fluorescent signal detected at the detector has intensity *I*(*x*). Therefore, the fluorescent intensity *I*(*x*) which is emitted from a fluorophore at a tissue depth *x* is described by:

1 $$I(x) = I_{0} \left[ {T_{at} \exp \left( { - \mu_{ex} x} \right)F\exp \left( { - \mu_{em} x} \right)T_{ta} } \right]$$

By assuming that *μ*_*T*_ = *μ*_*ex*_ + *μ*_*em*_ and *I*_*fluo*_ = *I*_*0*_*T*_*at*_*FT*_*ta*_, we can further simplify the model to:

2 $$I\left( x \right) = I_{fluo} \exp \left( { - \mu_{T} x} \right)$$

where *I*(*x*) is the intensity detected by the camera, *I*_*fluo*_ is the intensity of the fluorescent photon transmitting from the fluorophores without attenuation, and *μ*_*T*_ is the total tissue attenuation coefficient (Fig. 1B). Unlike some previous reports^7,27,32^, we do not explicitly separate the scattering contribution, in the form of the point spread function, from the attenuation term in this simplified model of light propagation. Rather we combine the effects of light scattering and tissue attenuation into a single exponential term. This assumption is consistent with the Lambert–Beer’s law and other 1D light propagation models in tissue^31,33,34^. Since we consider only the intensity change along a straight line between the camera system to the center of the fluorophore embedded in the tissue, there is only one spatial parameter in our model (*x*), which represents a distance from the surface (*x* = 0) to the fluorophore.

Next, for the fluorescent cell signal to be detected, the emitted photon intensity that reaches the camera, *I*(*x*), must be greater than or equal to a detection threshold, *T*, giving:

3 $${\text{Detect \, if }}I(x) \ge T$$

**Figure 2:**
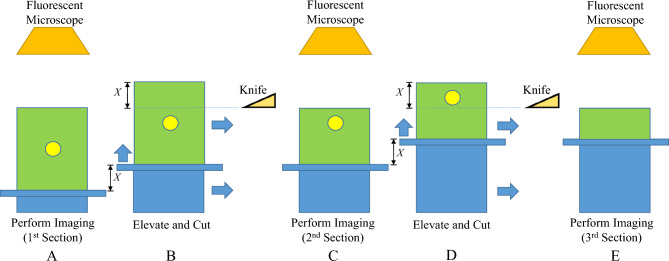
Slice-and-image operation in cryo-imaging. The diagram illustrates how the cryo-imaging acquires block-face images. a tissue sample (green box), containing a fluorescent cell (yellow circle), is fixed on a cutting stage (blue structures) that can move back and forth between the imaging position and the cutting position. After the first block-face image is taken by the camera above (**A**), the tissue sample is elevated by a fixed length of *X*, and then moved toward a sharp knife to cut off the top thin layer (with the thickness of *X*) (**B**). After the cut, the sample is moved back to the imaging position so that the second block-face image can be taken (**C**). The whole process of slice-and-image is repeated (**D**–**E**) until the whole sample is gone. Please note that this diagram is not to scale.

In cryo-imaging, the tissue sample is alternatively sliced and imaged with a fixed section thickness^1,2,4,10,15,18^. Diagrams in Fig. 2 illustrate how cryo-images are acquired. The tissue sample, containing a fluorescent cell, can move back and forth between the imaging position and the cutting position. The tissue sample can be elevated by a fixed length of *X*, and then moved toward a sharp knife to cut off the top thin layer. After the cut, the sample is moved back to the imaging position so that the block-face image can be taken. The whole process of slice-and-image is repeated until the whole sample is gone. Note that the fluorescent signal from a fluorescent cell deep within the sample can be dim at the block-face (Fig. 2A). However, as the sample is repetitively elevated, cut, and imaged, the cell intensity at the same location will be increasingly brighter in the output images (Fig. 2C). The cell signal will abruptly disappear when a tissue layer that contains the cell is cut (Fig. 2E). Therefore, signals of a single cell can be observed in multiple slices which results in a sub-surface fluorescence.

We introduce in the model a section thickness, *X*, which can vary from 1 to 300 µm. (For reference, in many experiments, we have set *X* between 20 and 40 µm for small animal imaging.) By considering Eqs. (2)–(3), there are four factors that contribute to the fluorophore signal detectability: the fluorophore signal *I*_*fluo*_ (gray level), the depth of the cell relative to the block-face surface *x* (μm), the tissue attenuation coefficient *μ*_*T*_ (cm^−1^), and the detection threshold *T* (gray level) (Fig. 1). Now consider the case that the fluorophores are evenly distributed throughout the tissue starting from the depth *x* = 0 (at the top edge) to *x* = *X* (at the edge of the section). According to Eq. (2), the fluorophores that give the least intensity are the ones residing at the bottom of the slice or at the depth *x* = *X*. Therefore, to be able to detect every fluorophore in the slice, the fluorophore intensity at the depth *x* = *X* must be at least the detection threshold. Hence, Eq. (3) becomes:

4 $$I_{fluo} \exp \left( { - \mu_{T} X} \right) = T$$

In this study, we define the optimal slice thickness *X*_*optimal*_ (μm) as the largest slice thickness that guarantees that the system can still detect the dimmest signals from fluorophores residing in the tissue slice. At depth *x* = *X*_*optimal*_, the fluorophores have a detected intensity equal to the detection threshold of the imaging system Eq. (4). By sectioning the tissue thicker than this value, the fluorophores at the lower edge of the slice will not be detected. By sectioning the tissue thinner than this value, all fluorophores can still be detected, but at the expenses of mechanical deteriorations, longer imaging time, and other costs. We can determine the optimal slice thickness by rearranging Eq. (4):

5 $$X_{optimal} = - \ln \left( {T/I_{fluo} } \right)/\mu_{T}$$

Interestingly, the model also suggests the optimal fluorescent intensity *I*_*optimal*_ (gray level) that guarantees the perfect detection of the fluorescent signals in the case that the slice thickness must be fixed. The optimal fluorescent intensity is the minimum intensity of the fluorescent cells that reside at the bottom of the slice (*x* = *X*) which can still be detected by the imaging system. At this intensity or greater, all fluorescent cells in the tissue slice are guaranteed to be detected. With the cells being dimmer than this value, the fluorescent signals at the bottom of the slice will be lost. With these definitions, Eq. (4) becomes:

6 $$I_{optimal} = T\exp \left( {\mu_{T} X} \right)$$

### Illustration of the effect of different section thicknesses on cell detectability

**Figure 3:**
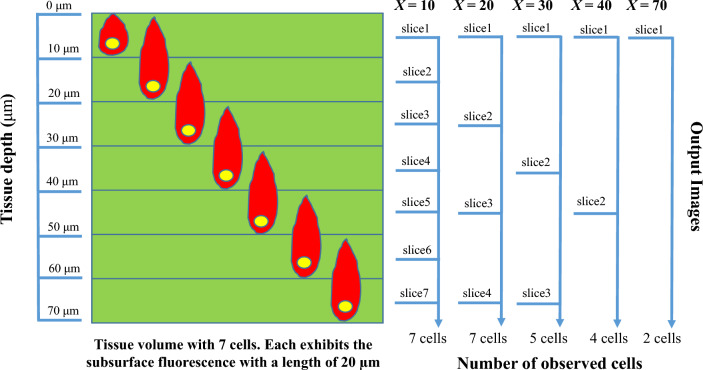
Illustration of the effect of different section thicknesses on number of cell counts. This example assumes 7 non-overlapping cells (yellow ovals) residing in each fixed depth interval of a tissue volume (green box). In this example, the depth interval (*t*) is 10 µm. We further assume that the cell signal can extend to the slice above, as the subsurface fluorescence, at most 10 µm before its signal becomes undetectable (red droplets). Thus, the subsurface fluorescent depth (*e*) is 20 µm. By sectioning this volume through the subsurface fluorescence signals, the corresponding cells are marked as detected in the output image slices. Multiple detections of the same cell can be grouped as one using the CCA algorithm. Therefore, by sectioning the volume with the section thickness (*X*) of 20 µm or less, one can resolve all cells inside the tissue. By sectioning it with larger section thicknesses, such as *X* = 30, 40, and 70 µm (above, right), one will miss the cells, which results in reduced detection sensitivity.

Figure 3 illustrates the interaction of slice thickness, fluorescence intensity, and cell counting. From the illustration, let us suppose that *N* fluorescent cells are evenly distributed in a homogeneous tissue volume with a total depth of *S* µm. Each cell is embedded in a non-overlapping fashion at a fixed tissue depth with an interval of *t* µm where *t* = *S*/*N*. Each cell has a subsurface fluorescence that can extend to multiple slices above with a length of *e* µm. During cryo-imaging, the tissue volume is alternately sectioned and imaged, with a section thickness of *X* µm. The process is repeated until the whole sample is gone. Approximately *S*/*X* fluorescent images are produced at the end of the process. To count the number of cells in the acquired volume, the connected component analysis (CCA) algorithm is applied to prevent multiple counting of the same cell due to subsurface fluorescence.

By slicing and imaging through the volume, the cell signals as well as the subsurface fluorescent signals can be used to resolve the number of cells in the volume. Figure 3 illustrates the situation where *S* = 70 µm, *N* = 7 cells, *t* = 10 µm, and *e* = 20 µm. With *X* = 20 µm, all cells in the tissue can be resolved and quantified correctly. Although, the distance between adjacent cells (*t* = 10 µm) is less than the slice thickness (*X* = 20 µm), the subsurface fluorescence of the skipped cell can still appear in the output image. By assuming that the signals of the adjacent cells do not overlap along the z-axis, the true number of cells can always be resolved. In the case of oversampling or using *X* < 20 µm, signals from the same cell can be detected multiple times, but with the help of CCA, numbers of cells can be resolved correctly. But in the range of under sampling or when *X* > 20 µm, the number of detected cells will be less than the true number of cells in the tissue as shown in Fig. 3. This situation leads to false negatives. Next, we will propose a mathematical model of the relationship between the fluorescent cell detectability and the slice thickness especially in the sub-optimal range (*X* > *X*_*optimal*_).

### Mathematical relationship between detection sensitivity and slice thickness

In the sub-optimal range (*X* > *X*_*optimal*_), the number of detected cells decreases as the slice thickness increases. By formulating the mathematical relationship, one can predict the cell loss and even estimate a correction factor to compensate for the under-sampling. In this section, we aim to construct the relationship under some simplifications.

Recall that fluorescent signals of the same cell can be observed in multiple slices as a subsurface fluorescence (Figs. 3 and 5). The out-of-plane fluorescence gives a subsurface haze in 2D images and an elongation artifact along the z-axis that appears as a “comet tail” in 3D^7,30,34^. The length of the signal from a single fluorescent cell is governed by Eqs. (2) and (3). If the imaging system slices through any part of the subsurface fluorescence, the cell signal should appear in the output image. Therefore, this subsurface fluorescent length (*e*) is indeed the optimal slice thickness (*X*_*optimal*_) as illustrated previously. By considering *X* > *X*_*optimal*_ (which implies *e*/*X* < 1.0), the number of observed cells (*n*) is reduced from the total cells in the sample (*N*) according to the relative distribution, *d*(*x*), of cells lying in a given slice. The distribution of cells is therefore also related to the probability of a cell being at a given distance, *p*(*x*). This can be formulated as

7 $$n = \int_{0}^{X} {d(x)dx} = N\int\limits_{0}^{x} {p(x)dx}$$

8 $$n = \int_{0}^{X} {d(x \le X_{optimal} )dx} = \int_{0}^{{X_{optimal} }} {d(x)dx} = N\int_{0}^{{X_{optimal} }} {p(x)dx}$$

Note that the probability distribution, *p*(*x*), for the slice thickness *X* is determined from the distribution within each of those slices, *d*_*i*_(*x*). If we consider that there could be varying distributions across slices, then the average *p*(*x*) would be determined as below.

9 $$p(x) = \frac{{\sum\nolimits_{i}^{M} {d_{i} (x)} }}{N} = \frac{{\sum\nolimits_{i}^{M} {N_{i} p_{i} (x)} }}{N} = \sum\limits_{i}^{M} {w_{i} p_{i} (x)}$$

where *M* is the number of slices, *i* is the slice index, *w*_*i*_ = *N*_*i*_/*N*.

The detection sensitivity (*Sens*) is given by the ratio of *n*/*N*, which is dependent only on the probability distribution of fluorescent cells,

10 $$Sens = \frac{n}{N} = \frac{{N\int_{0}^{{X_{optimal} }} {p(x)dx} }}{{N\int_{0}^{X} {p(x)dx} }} = \frac{{\int_{0}^{{X_{optimal} }} {p(x)dx} }}{{\int_{0}^{X} {p(x)dx} }}$$

Assuming a uniform probability density function that would correspond to randomly distributed cells in a large tissue region, the sensitivity can be formulated as follows:

11 $$n = \left\lceil {\frac{e}{X}N} \right\rceil$$

As such, the sensitivity is linearly proportional to the reciprocal of *X*, and the length of the subsurface fluorescence (e):

12 $$Sens \propto \frac{e}{X}$$

By taking the logarithm on both sides and replacing *e* with *X*_*optimal*_, it reveals a perfect -45º linear relationship that can be formed between log(*Sens*) vs. log(*X*):

13 $$\log \left( {Sens} \right) = - \log \left( X \right) + \log \left( {X_{optimal} } \right)$$

Recall that beyond the optimal slice thickness (*X* > *X*_*optimal*_), the detection sensitivity decrease as the slice thickness increases. Also, remind that for all *X* values that are less than or equal to *X*_*optimal*_, the *Sens* value will be 100%. By combining *X* values from both ideal range (*X* ≤ *X*_*optimal*_) and sub-optimal range (*X* > *X*_*optimal*_), we obtain:

14 $$Sens = \left\{ {\begin{array}{*{20}c} {100\%, } & {X \le X_{optimal} } \\ {\frac{{X_{optimal} }}{X},} & {X > X_{optimal} } \\ \end{array} } \right.$$

Relationships derived in Eq. (14) can be used to estimate the missing fluorescent cells in the image data if one performs cryo-imaging with a slice thickness larger than the optimal value. By definition of sensitivity, the true number of cells, *Count*(*X*_*optimal*_), can be estimated by dividing the number of observed cells in the volume, *Count*(*X*), with the sensitivity, *Sens*(*X*):

15 $$Count\left( {X_{optimal} } \right) = \frac{Count\left( X \right)}{{Sens(X)}}$$

To this end, the previous relationship is established under the following key assumptions:

- All cells are embedded in a homogeneous tissue: Variance(*μ*_*T*_) ≈ 0.
- The cells in the tissue have the same intensity: Variance(*I*_*fluo*_) ≈ 0.
- The cells are regionally well-distributed in the tissue with no overlap.

In the case that there is some distribution, *D*(*I*_*fluo*_), that pertains to the *N* cells, then overall sensitivity is given by:

16 $$Sens = \frac{{\int_{0}^{\infty } {Sens(I_{fluo} )D(I_{fluo} )dI_{fluo} } }}{{\int_{0}^{\infty } {D(I_{fluo} )dI_{fluo} } }} = \frac{{\int_{0}^{\infty } {Sens(I_{fluo} )D(I_{fluo} )dI_{fluo} } }}{N}$$

which, when considering that the cells have at most *K* different intensity values, can be simplified to a discretized summation

17 $$Sens = \frac{{\sum\nolimits_{j}^{K} {Sens(I_{fluo,j} )N_{j} } }}{N} = \sum\limits_{j}^{K} {\frac{{N_{j} }}{N}} Sens(I_{fluo,j} )$$

where *j* is an index corresponding to the single intensity *I*_*fluo, j*_ pertaining to *N*_*j*_ cells and *K* is the number of unique *I*_*fluo*_ values. Note that *X*_*optimal*_ is non-negative only for values of *I*_*fluo*_ ≥ *T*, but *Sens*(*I*_*fluo*_) = 0 for any *I*_*fluo*_ values below *T*. In a case where cells have *I*_*fluo*_ < *T*, those cells will not be detected independently of slice thickness, and therefore the excitation intensity *I*_*0*_ needs to be increased or other components along the fluorescence imaging chain need to be improved beyond the consideration of this work.

Next, we will show that the above equations conform with real data by performing in silico experiments and comparing the results with real data. Later, we will discuss additional in silico experiments in the situations where the key assumptions are not met.

## Experiments

### Digital sectioning simulation of model cells

We programmatically implemented digital sectioning simulations to validate the relationship in Eqs. (13)–(14). These in silico experiments were built upon the model illustrated in Figs. 1, 2, 3 to validate the relationship between slice thickness and detection sensitivity derived earlier. The digital sectioning simulation algorithm is summarized as follows: (1) Create a virtual homogeneous volume of whole sample (thickness = *S* μm) and an effective attenuation coefficient (*μ*_*T*_, cm^−1^), (2) Distribute model cells at every 1 μm of the volume. Since there is one cell per 1 μm depth, there will be *N* = *S* cells in the whole volume. Each cell has a grayscale intensity of *I*_*fluo*_, (3) For each cell residing in the volume, we measure *I*(*x*) at the surface of the block-face where *x* is the depth of the cell relative to the block-face. The value of *I*(*x*) is determined by a governing equation in Eq. (2), (4) If *I*(*x*) is greater than or equal to the detection threshold, *T*, then the testing cell will be marked as detected [Eq. (3)]. (5) Section away the top layer of the volume by *X* μm, (6) Repeat step (3)–(5) until the whole sample volume is completely removed, (7) Output the number of detected cells. Details of the algorithm are described in Pseudocode 1.
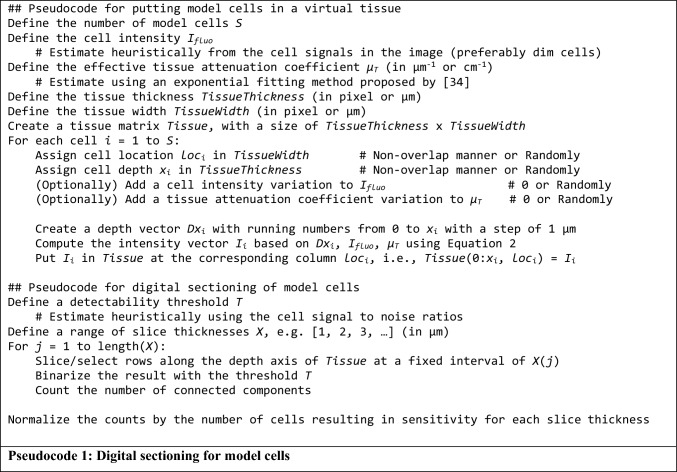


With the algorithm, we conducted two in silico experiments with parameter values in the range typically observed in cryo-imaging applications. For the first experiment, we varied section thickness and measured the corresponding sensitivity: *Sens* = *n*/*N* = TP/(TP + FN) where TP, FP, and FN are true positives, false positives, and false negatives, respectively. We expect that sectioning the sample thicker than the optimal section thickness (*X* > *X*_*optimal*_) would adversely affect the sensitivity. Three cell intensities were chosen for this simulation: *I*_*fluo*_ = 30, 50 and 80 (grayscale intensity). The numbers were chosen from the intensity distribution of cells derived from our stem cell imaging database. Parameters *T* and *μ*_*T*_ were set to 10 (gray level) and 314 cm^−1^, respectively. The detection threshold value (*T*) was empirically estimated based on the signal-to-noise-ratio (SNR) in our image data. The value of *μ*_*T*_ was estimated from mouse tissues in our database using an exponential fitting method proposed by Steyer et al.^34^. The programming platform used in this study was Matlab 2022a (MathWorks, USA).

In the second experiment, we fixed the slice thickness but varied the cell intensity and measured corresponding sensitivity. By fixing slice thickness (*X* = 20 μm), we expect that cells with intensity below the optimal intensity (*I*_*fluo*_ < *I*_*optimal*_) could lead to lower detection sensitivity. Three different tissue attenuation coefficients were chosen, *μ*_*T*_ = 214, 314 and 414 cm^−1^, with an increment of 100 cm^−1^. These values were consistent with the literature^33,34^. Other used parameters were *T* = 10 and *X* = 20 μm. This experiment aimed to show the effect of different tissue attenuations on detection sensitivity.

### Section-and-image simulation of the real data

To further test the validity of our model, we compared the simulated results to real data. We performed the section-and-image operation on the real data to generate thicker sections. These new sections were taken from thinly sliced real images that contained fluorescent cells. There were two datasets that we used in this study: (1) fluorescently labeled stem cells in a mouse model^2,9,11,12^, and (2) fluorescent microspheres in a pig model^5,26^. Details about the animal experiments are described in the Supplemental Document. All animal experiments were performed in accordance with relevant guidelines and regulations. They were approved by the Institutional Animal Care and Use Committee (IACUC) at Case Western Reserve University. We also confirm that the study is reported in accordance with ARRIVE guidelines. Next, we describe steps to simulate thick sections taken from thinly sliced real data to determine the effect of the slice thickness on the detection sensitivity. We hypothesize that results predicted by the theory [Eqs. (13)–(14)] should correlate with real data.

**Figure 4:**
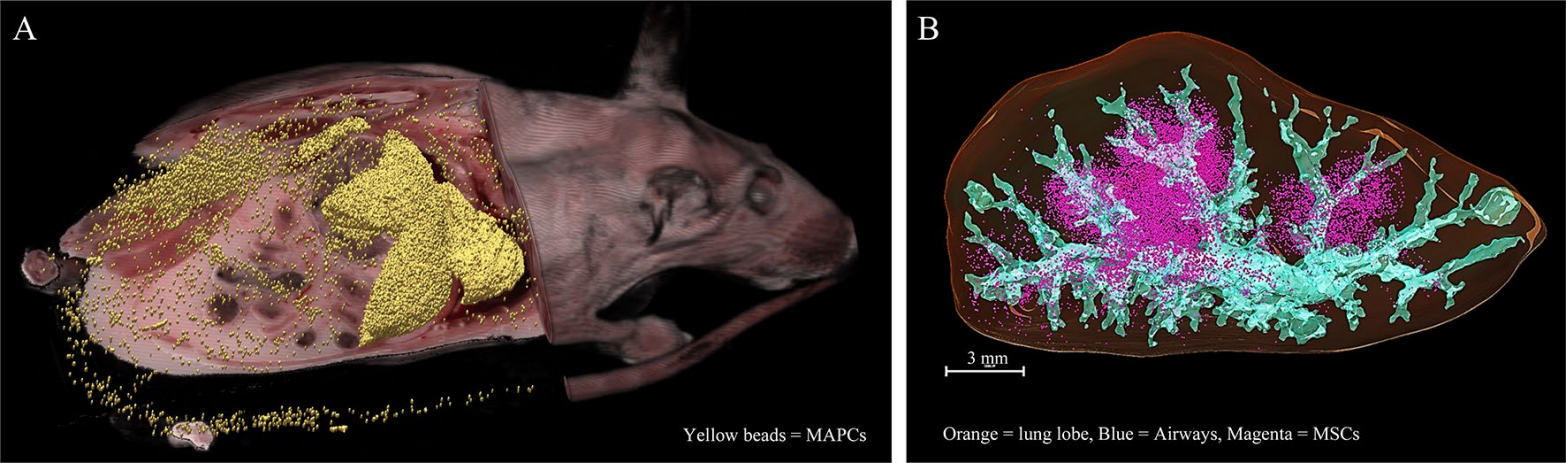
Cryo-imaging enabled visualization and quantification of stem cells anywhere in mice with very high recovery rate. A mouse was intravenously injected with 5 × 10^5^ stem cells. Approximately 290,000 cells were detected and visualized (yellow beads in **A**). The recovery rate (number detected cells/delivery) was 58%. In another experiment, 1 × 10^5^ stem cells were directly injected into a lung lobe of a mouse. About 94,269 cells were detected (magenta beads in **B**). This yielded 94% recovery rate.


In the first dataset, we used the stem cell dataset for simulation. A raw image zooming to the fluorescent cell signals is shown in Suppl. Fig. 1A. The images were then stacked, registered, and visualized in 3D in Fig. 4B. To perform section-and-image simulations, we created a new volume of a thicker slice data by choosing images from the original volume but skipping every 2 slices. Since the section thickness was originally set to 20 μm, the new volume, which consisted of half of the original images, would have a slice thickness of 40 μm. To obtain volumes of different slice thicknesses, we repeated the experiment by skipping every 3, 4, 5 …, 50 slices to resemble cryo-imaging with the slice thickness of 60, 80, 100, …, 300 μm, respectively. For each volume, the number of stem cells were detected and quantified using the algorithm previously published, which includes 3D CCA^10^. We summarize the process in Pseudocode 2. To compare the results with our model, we ran an in silico simulation (Pseudocode 1) with parameters estimated from the real data. Briefly, *I* was estimated using the mean intensity of detected cells. *T* was heuristically estimated by optimizing the number cell detections while minimizing the false positives, and finally *μ*_*T*_ was estimated using an exponential regression method proposed by Steyer et al.^34^. To test the validity of the simulation, we performed a correlation testing on the simulation results with the results from real data. We employed Pearson product-moment correlation coefficient and root-mean-squared error (RMS error) as the metrics.

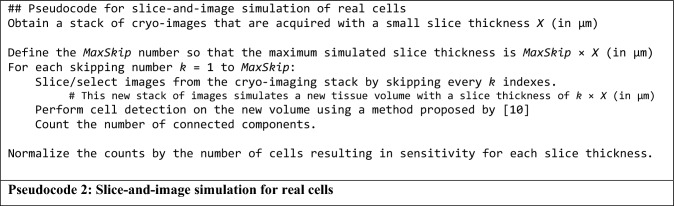


**Figure 5:**
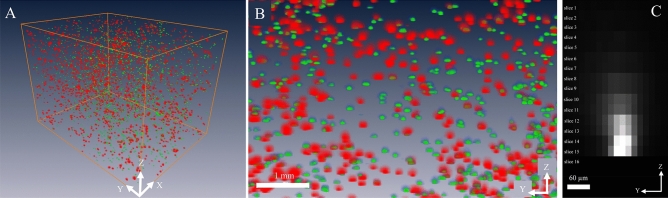
Red and green microspheres in the pig myocardium cryo-imaging data. Red microspheres were injected into the left ventricle of a pig during a baseline condition while green microspheres were injected to the same site during induced ischemia. Figures (**A**) (zoom out) and (**B**) (zoom in) show volume renderings of the microspheres entrapped in the myocardium tissue from an apex segment of the heart. The fluorescent signals were false-colored according to the microsphere emission spectra (red and green). Notice that the red microspheres were brighter than the green microspheres under our imaging system. Figure (**C**) shows subsurface fluorescence of a red microsphere which extended several slices above the slice that contains the microsphere (slice 15). Once the slice was sectioned, the signal disappeared (slice 16). In this example, the intensity in the slices beyond ‘slice 10’ were below the detectability threshold. The slice thickness was 10 µm.

In the second dataset, we used images of a pig heart, containing fluorescent microspheres, to perform the simulation. Red and green microspheres were injected into the pig’s right ventricle under different physiological conditions (see Supplemental Document). Since the pig heart was quite large, we used only a small portion of the tissue. The section thickness was set to 10 μm. Because red microspheres and green microspheres had different brightness under our standard imaging filter^1,2,5,9^, we processed them separately. The red microspheres were of much brighter intensity than the green microspheres. Examples of the fluorescent microsphere signals and the 3D visualization are shown in Suppl. Fig. 1B and Fig. 5, respectively. We performed the section-and-image simulation to create new volumes using the same process as that applied to stem cell data. The parameters *I*_*fluo*_, *T*, and *μ*_*T*_ for the microspheres were also estimated using the same method described previously. Again, the in silico results were compared against real data.

### In silico simulations for non-compliant situations

To show the effects of non-compliances in the key assumptions to the detectability, we performed 3 additional in silico simulations. The simulations aim to study the effects of (1) tissue inhomogeneity, (2) cell brightness variations, and (3) cell signal overlapping on the detectability as observed in the sensitivity-thickness curves. In the first simulation, the model cells were placed in a virtual tissue in a non-overlapping fashion. All cells had the same brightness (*I*_*fluo*_ = 40) but experienced different tissue attenuations *µ*_*T*_ to simulate situations where the tissue of interest is a mix of different tissue types (inhomogeneous tissue). Four levels of variability were tested: (1) *µ*_*T*_ = 314 cm^−1^ (no variation), (2) *µ*_*T*_ = 314 ± *N*(σ ~ 50) cm^−1^, (3) *µ*_*T*_ = 314 ± *N*(σ ~ 100) cm^−1^, and (4)* µ*_*T*_ = 314 ± *N*(σ ~ 150) cm^−1^, where *N*(σ) represents a normal random number generator with zero mean and a standard deviation of σ. The digital sectioning was performed to measure the sensitivity vs. the slice thickness according to the Pseudocode 1.

In the second simulation, the model cells were placed in a virtual homogeneous tissue but with variations of the cell brightness. Four levels of variability were tested: (1) *I*_*fluo*_ = 40 (no variation), (2) *I*_*fluo*_ = 40 ± *N*(σ ~ 5) cm^−1^, (3) *I*_*fluo*_ = 40 ± *N*(σ ~ 10) cm^−1^, and (4) *I*_*fluo*_ = 40 ± *N*(σ ~ 15) cm^−1^. The effective tissue attenuation coefficient *µ*_*T*_ was fixed at 314 cm^−1^. The variability may represent situations such as cells losing fluorescent dyes, cell dead, dividing cells, photobleaching, poor labeling, etc. Then, we performed the digital sectioning on these simulations to measure the sensitivity-thickness curve as described in the Pseudocode 1.

In the last simulation, we simulated the effect of different degrees of cell overlapping to the detectability. Cell overlapping is defined as two different cells that reside closely to each other along the depth axis such that their sub-surface fluorescent signals may merge and be ‘seen’ as a single signal. This may cause a decrease in the detection sensitivity as two or more cells are only counted as one. To show the effect, the simulation was performed by randomly placing the model cells on a fixed tissue area to allow the subsurface fluorescence’s of multiple cells to overlap. The degree of overlapping is governed by *Density* which is calculated by the number of model cells to tissue area (in the unit of cells/cm^2^). Four degrees of cell overlapping were tested: (1) No overlap, (2) *Density* = 400 cells/cm^2^, (3) *Density* = 800 cells/cm^2^, and (4) *Density* = 1600 cells/cm^2^. The values of *µ*_*T*_ and *I*_*fluo*_ were fixed at 314 cm^−1^ and 40, respectively. The different degrees of cell overlapping represent cases like immune cell aggregation in the lymphoid/inflamed tissues, tumor masses, pulmonary emboli, etc. Again, the sensitivity-thickness curve was measured and reported.

## Results

Cryo-imaging enabled 3D visualization and quantification of fluorescently labeled cells throughout a mouse with a very high recovery rate. As described in the Supplemental Document, the mouse was intravenously injected with 5 × 10^5^ MAPCs. After running the stem cell detection algorithm^10^, approximately 290,000 cells were detected and visualized (yellow beads in Fig. 4A). In this dataset, the recovery rate (number detected cells/delivery) was 58%. In another experiment, 1 × 10^5^ FAC-sorted MSCs were directly injected into a lung lobe of a mouse. After applying the algorithm, 94,269 cells were detected (magenta beads in Fig. 4B). This yields a 94% recovery rate. Number of detections in mice injected with unlabeled cells (false positives) were significantly small (p < 0.01, two tailed Student’s t-test). Examples of stem cell signals are shown in Suppl. Fig. 1A.

**Figure 6:**
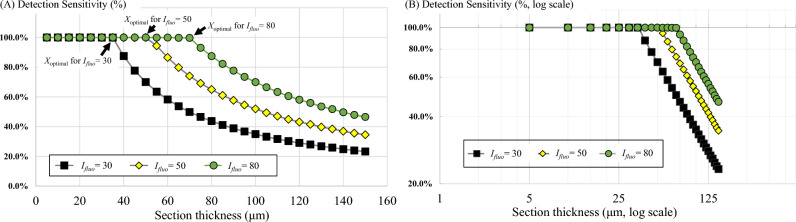
The relationship between section thickness and detection sensitivity estimated by the simulation was consistent with the theory. The simulation result in (**A**) reveals the optimal slice thickness (*X*_*optimal*_) which is the largest slice thickness that yields 100% detection sensitivity. By sectioning the volume with a section thickness larger than this value (toward the right of the graph), the sensitivity decreased significantly. In this simulation, we simulated 3 levels of cell intensity: *I*_*fluo*_ = 30, 50, and 80 (gray level). We observed that the brighter the fluorescent cells were, the higher the value of the slice thickness. This was consistent with our derivation in Eq. (14). Interestingly, when we plotted the relationship in a log–log scale (**B**), the graph showed perfect linearity over the sub-optimal range (*X* > *X*_*optimal*_).

**Figure 7:**
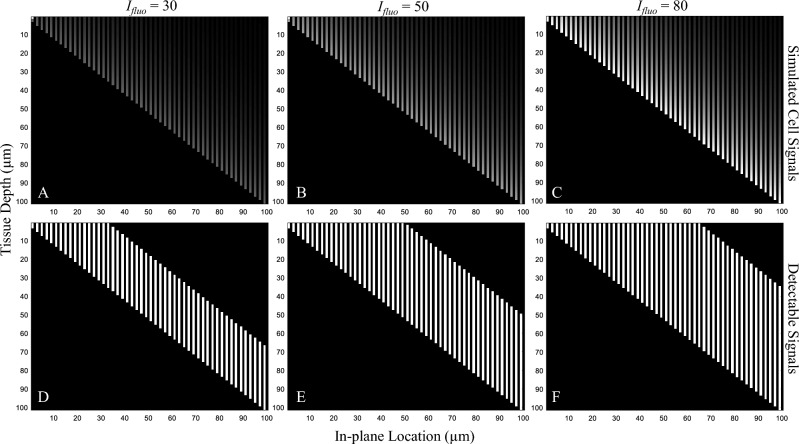
Illustration of the effect of different cell intensities on the detectability. We simulated situations with 3 cell intensities: *I*_*fluo*_ = 30 in (**A**), 50 in (**B**), and 80 in (**C**). In the simulations, we placed synthetic cells in a non-overlapping fashion in a virtual volume. Each cell extended its subsurface fluorescence upward as governed by Eq. (2). Detectability visualization of the cells in (**A**–**C**), were shown in (**D**–**F**), respectively. These were performed by binarizing the cell signals in (**A**–**C**) using Eq. (3). The results show that the cell detectability (the sub-surface fluorescence length) is linearly proportional to the cell intensity.

The in silico simulations revealed the relationships between slice thickness and detection sensitivity. We performed two simulations—in the first one, we performed digital sectioning with different slice thicknesses and measured the corresponding detection sensitivity. The slice thickness (*X*) ranged from 5 to 150 μm. We observed that with small values of slice thickness, the sensitivity remained at 100% until the value reached a certain point at which the sensitivity started to decrease (Fig. 6). This point was indeed the optimal slice thickness (*X*_*optimal*_) for cryo-imaging. In this simulation, we examined three intensity values: *I*_*fluo*_ = 30, 50, and 80 (gray level). The parameters *T* and *μ*_*T*_ were set to 10 (gray level) and 314 cm^−1^, respectively. We illustrated the simulations in Fig. 7 to show how the model cell signals and their detectability appear in the virtual volume. Note that the cells were placed in the tissue in line with the non-overlapping assumption. As a result, we found that *X*_*optimal*_ for *I*_*fluo*_ = 30, 50 and 80 simulations were 35 μm, 51 μm and 66 μm, respectively. The results were consistent with the optimal section thickness estimated by Eq. (5). We observed that for lower fluorescent intensity levels (*I*_*fluo*_), the sensitivity decreased at a higher rate (different markers in Fig. 6A). In the sub-optimal range (*X* > *X*_*optimal*_), the curve appeared as the reciprocal function derived in Eq. (14). By showing the relationship on a log–log scale, the relationship is shown to be perfectly linear (Fig. 6B), as predicted by the mathematical derivation described in Section “In silico simulations for non-compliant situations”.

**Figure 8:**
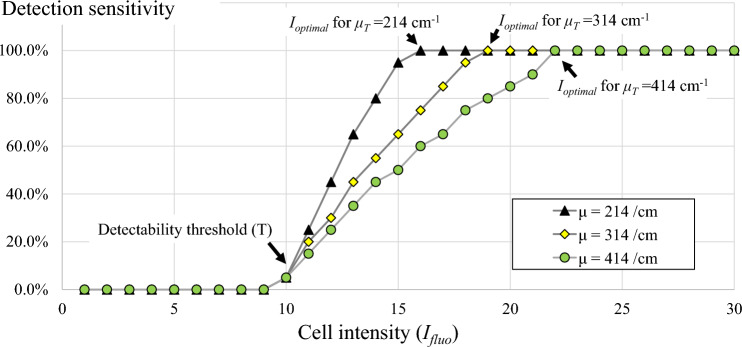
The relationship between cell intensity and detection sensitivity estimated by the simulation was also consistent with the theory. In this in silico simulation, the detection sensitivity was recorded for each change in cell brightness. The result suggests that in order to maintain 100% detection sensitivity, the cell intensity must at least the optimal cell intensity as predicted in Eq. (6). On the other hand, with the intensity being smaller than this value (toward the left of the x-axis), the sensitivity decreased significantly. By increasing the tissue attenuation coefficient (*μ*_*T*_), one would need higher cell intensity to maintain 100% detection sensitivity (different markers).

**Figure 9:**
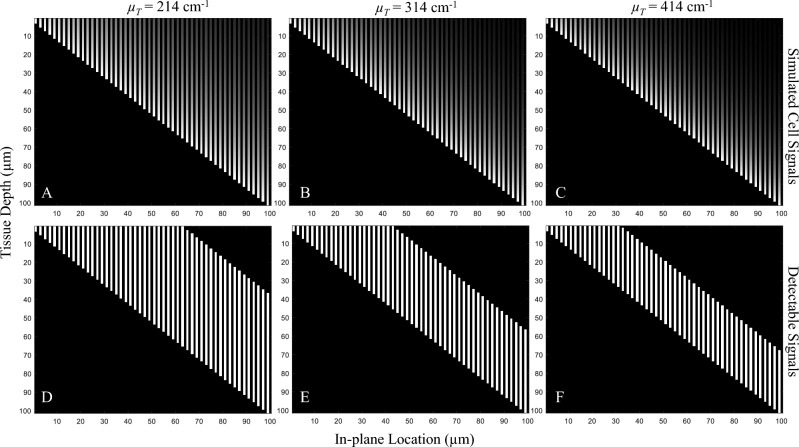
Illustration of the effect of different tissue attenuation coefficients on the detectability. We simulated situations with 3 tissue attenuation coefficients: *µ*_*T*_ = 214 cm^−1^ in (**A**), 314 cm^−1^ in (**B**), and 414 cm^−1^ in (**C**). Detectability visualization of the cells in (**A**–**C**), were shown in (**D**–**F**), respectively. The results show that the cell detectability (the sub-surface fluorescence length) is inversely correlated to the tissue attenuation coefficient as described in Eq. (5).

For the second simulation, we studied the effect of cell intensity levels on the detection sensitivity (Fig. 8). In this simulation, we fixed the section thickness (*X* = 20 μm) but varied the cell intensity ranging from 1 to 30 (gray level). We also repeated the simulation with different tissue attenuation coefficients (*μ*_*T*_) which were 214, 314, and 414 cm^−1^ (steps of 100 cm^−1^). The result in Fig. 8 shows that the sensitivity could be maintained at 100% when the cell intensity was greater than the optimal intensity as predicted in Eq. (6). We found that for *μ*_*T*_ = 214, 314 and 414 (cm^-1^), the estimated values of *I*_*optimal*_ were 15.34, 18.74 and 22.89 (gray level), respectively. The result shows that the higher the values of *μ*_*T*_ were, the higher the cell brightness required for maintaining 100% sensitivity. As expected, cells with the intensity below the detectability threshold (*T* = 10) resulted in 100% loss (0% sensitivity). We also illustrated the simulations in Fig. 9 to show how the model cell signals and their detectability appear in the virtual volume. The results show that the cell detectability (the sub-surface fluorescence length) is inversely correlated to the tissue attenuation coefficient as described in Eq. (5).

**Figure 10:**
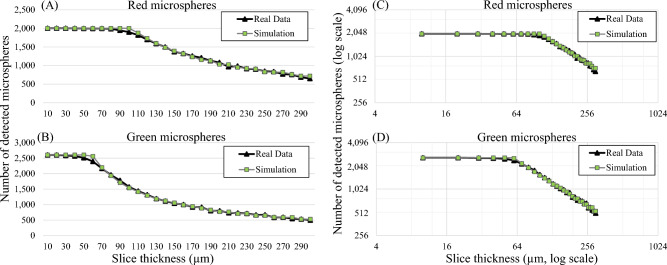
Microsphere detectability vs. slice thickness relationship obtained from real data were highly correlated with that from the simulations. In this experiment, the tissue volume containing about 4600 fluorescent microspheres was sectioned with different slice thickness (from 10 to 300 µm). Number of microsphere detections for each slice thickness is shown (black line with triangle markers) for red microspheres in (**A**) and for green microspheres in (**B**). With carefully chosen parameters, our simulation could predict the relationships that completely matched the real data (gray line with square markers). Interestingly, when we plot the relationships on a log–log scale (**C**–**D**), the results show the perfect linear relationship between the number of detections and the slice thickness. This could be used to estimate the true number of cells in the data, especially when the chosen slice thickness is larger than optimal.

We next discuss the correlation between our simulations and real data. For the pig myocardial tissue data, we found about 4600 fluorescent microspheres (red and green) in the volume of interest (Fig. 5). We performed section-and-image simulation with different slice thicknesses ranging from 10 to 300 μm. After performing microsphere quantification, the number of red and green microspheres are shown in Fig. 10A and B, respectively. As expected, with small values of slice thickness (toward the left of the graph), all microspheres in the tissue volume could be resolved correctly. By increasing the slice thickness value (towards the right of the graph), the sensitivity could only be maintained at 100% until the value reached a certain point (*X*_*optimal*_) at which the false negatives start to increase. This observation held true in both red and green microsphere datasets. We independently ran in silico simulations creating the relationships based on estimated parameters. The parameter sets were found to be (*I*_*fluo*_ = 300,* μ*_*T*_ = 332 cm^−1^) for the red microsphere simulation, and (*I*_*fluo*_ = 87, *μ*_*T*_ = 372 cm^−1^) for the green microsphere simulation, respectively. The results in Fig. 10 show that the slice thickness/detectability relationship from the simulation were nearly matched to the real data. The correlation coefficients between the two relationships were 0.998 and 0.999 for the red and the green microspheres, respectively. After detecting and quantifying the microspheres, the dataset contained about 2000 red microspheres and 2600 green microspheres. The measured RMS errors were 36.54 (1.82%) and 42.87 (1.65%) for the red and the green microspheres, respectively. The results also show that the estimated values of *X*_*optimal*_ were 102.45 μm for the red microspheres and 58.15 μm for the green microspheres. These values indicate the best section thickness that guaranteed ideal microsphere detection with the minimum number of cuts. We observed that the red microspheres were approximately 3 times brighter than the green microspheres in the raw data (Suppl. Figs. 1B and Fig. 5). As the result, the red microspheres allowed larger slice thickness as compared to the green microspheres (Fig. 10A,B). This was consistent with our derivation in Eq. (5). Again, by plotting the relationships on a log–log scale, the results show near-perfect linear relationships in the sub-optimal range (*X* > *X*_*optimal*_) (Fig. 10C,D).

**Figure 11:**
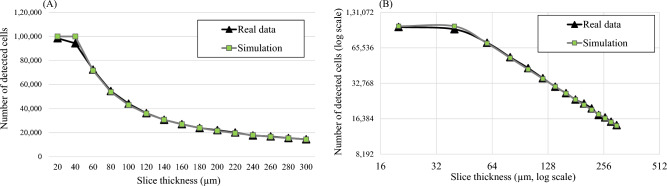
The cell detectability vs. slice thickness relationship obtained from real data were also highly correlated with that from the simulations. In this experiment, a mouse lung was injected with 1 × 10^5^ fluorescently labeled stem cells. The tissue volume was sectioned with different slice thicknesses (from 20 to 300 μm). The numbers of cell detection for each slice thickness are reported (**A**). The relationship on a log–log scale is also shown in (**B**). Again, the simulation could predict the relationship as it nearly overlapped (gray line with square markers) with the real data (black line with triangle markers). The correlation coefficient between real and simulated data was 0.999. *X*_*optimal*_ value was around 40 μm where the model predicted the *X*_*optimal*_ value to be 42.52 μm.

The relationship obtained from the stem cell dataset was also consistent with the simulation. In this dataset, about 1 × 10^5^ fluorescently labeled stem cells were directly injected into a lobe of the mouse lung. Simulated section-and-image and cell detection were repeatedly performed on the tissue volume with different slice thickness ranging from 10 to 300 μm. The results in Fig. 11 show that the “cell detection vs. slice thickness” relationship was consistent with the theory. The relationship obtained from the simulation was also well-matched to real data. The correlation coefficient was 0.999 and the RMS error was 1601 cells (or 1.6% of the injected cells). The results in Fig. 11 suggest that the *X*_*optimal*_ was around 40 μm where the model predicted the *X*_*optimal*_ to be 42.52 μm. The estimated parameters were *T* = 10, *I*_*fluo*_ = 38 and *μ*_*T*_ = 314 cm^−1^ for the red quantum dot labeled stem cells. We observe that the range of the *X*_*optimal*_ values for the cells was much narrower than that of the fluorescent microspheres (~ 40 μm vs. ~ 60–100 μm). This was because the cell brightness (~ 40) was much less than that of the microspheres (~ 80–400).

**Figure 12:**
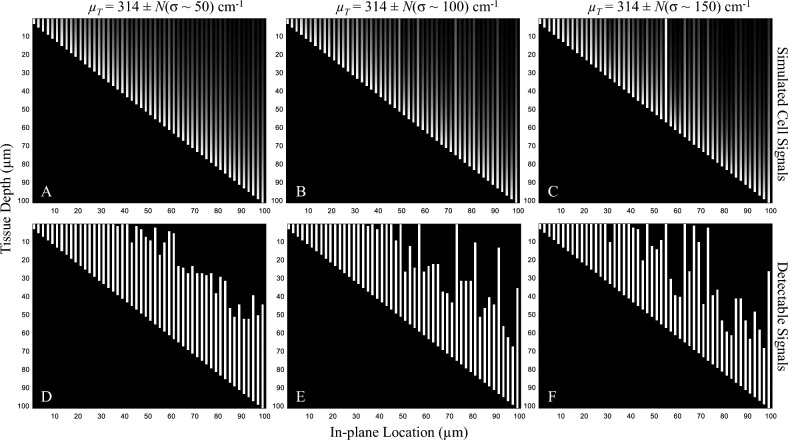
Illustration of the effect of the tissue inhomogeneity on the detectability. The *µ*_*T*_ in simulations (**A**–**C**) were set to 314 ± *N*(σ ~ 50) cm^−1^, 314 ± *N*(σ ~ 100) cm^−1^, 314 ± *N*(σ ~ 150) cm^−1^, respectively. *N*(σ) represents a normal random number generator with zero mean and a standard deviation of σ. Detectability visualization of the cells in (**A**–**C**), were shown in (**D**–**F**), respectively. The higher value of σ reflects the higher level of inhomogeneity in the tissue attenuation coefficient.

**Figure 13:**
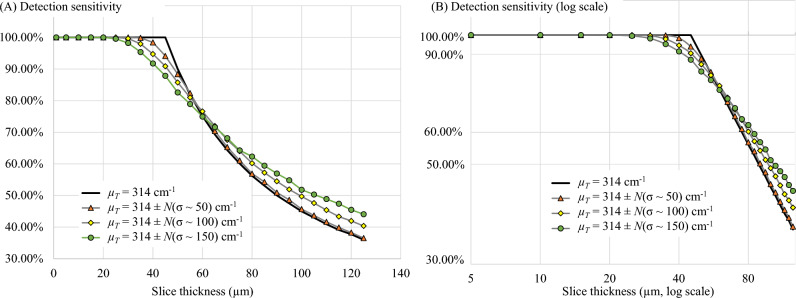
The effect of the tissue inhomogeneity on the detection sensitivity. With no variation such as in a homogeneous tissue [black line in (**A**)], the detection sensitivity starts to decrease exactly at the optimal slice thickness point. With the variations such as in non-homogeneous tissues, the sensitivity decreased at much earlier than the optimal point. The higher the variation is, the less steep of the sensitivity-thickness slope (lean toward 0°) in the log–log scale (**B**).

Additionally, we conducted three in silico experiments to show the effects of key assumptions on the detectability. First, the tissue inhomogeneity effect was tested by assigning the model cells with different *µ*_*T*_ values to simulate situations where the tissue of interest is a mix of different tissue types. Figure 12 illustrates the cell signals and their detectable signals in three virtual tissues, each with different levels of *µ*_*T*_ variability. Please note that the cell intensity and their detectability in all results were governed by Eqs. (2) and (3), respectively. The corresponding sensitivity-thickness curves are shown in Fig. 13. As compared to the homogeneous tissue, the sensitivity in the non-homogeneous tissue decreased earlier than the optimal point. The optimal points in these situations are not well-defined and hard to determine in the sensitivity-thickness curve. We also observe that the higher the variation is, the less steep of the sensitivity-thickness slope (lean toward 0°) in the log–log scale (Fig. 13B).

**Figure 14:**
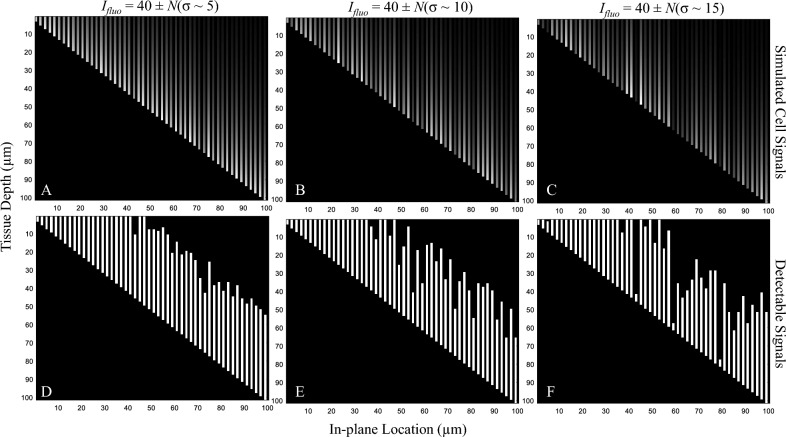
Illustration of the effect of the cell brightness variations on the detectability. The *I*_*fluo*_ in simulations (**A**–**C**) were set to 40 ± *N*(σ ~ 5), 40 ± *N*(σ ~ 10), 40 ± *N*(σ ~ 15), respectively. *N*(σ) represents a normal random number generator with zero mean and a standard deviation of σ. The higher value of σ reflects the higher level of inhomogeneity in the cell brightness. Detectability visualization of the cells in (**A**–**C**), were shown in (**D**–**F**), respectively.

**Figure 15:**
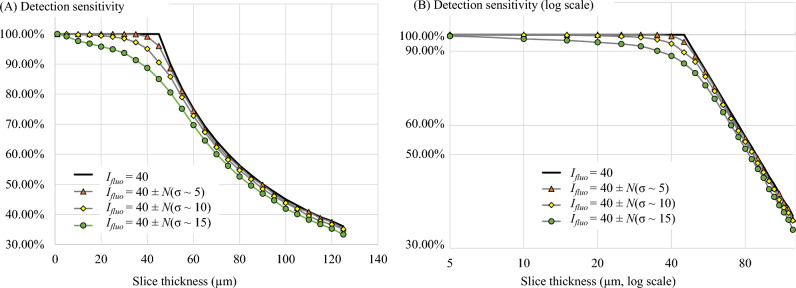
The effect of cell brightness variations on the detection sensitivity. With no variation in the cell intensity (black line in (**A**)), the detection sensitivity starts to decrease exactly at the optimal slice thickness point. With the variations, the sensitivity decreased at much earlier than the optimal point. The optimal points in these situations are not well-defined and hard to determine in the sensitivity-thickness curve. We also observe that all sensitivity-thickness curves maintained the same slope at − 45° in the log–log scale (**B**).

Second, the cell brightness variation effect was tested by assigning the model cells with different *I*_*fluo*_ values. Figure 14 illustrates the cell signals and their detectable signals in three virtual tissues, each with different levels of *I*_*fluo*_ variability. The corresponding sensitivity-thickness curves are shown in Fig. 15. As compared to the stable cell brightness case, the sensitivity decreased at much earlier than the optimal point. Again, the optimal points are not well-defined and hard to determine in the sensitivity-thickness curve. Additionally, we observe that all sensitivity-thickness curves maintained the same slope at − 45° in the log–log scale (Fig. 15B).

**Figure 16:**
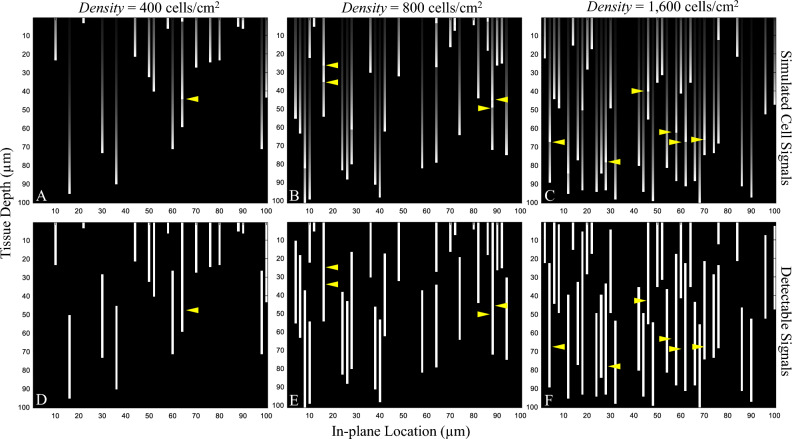
Illustration of the effect of the cell overlapping on the detectability. Any two cells can overlap one another if they are too closed along the vertical axis such that they were ‘seen’ as one object (yellow arrow heads). The overlapping effect always results in false negative or missing of cells. We also experimented with different cell density to exaggerate the cell overlapping effects. In simulations (**A**–**C**), the cell densities were set to 400, 800, 1600 cells/cm^2^, respectively. Detectability visualization of the cells in (**A**–**C**), were shown in (**D**–**F**), respectively.

**Figure 17:**
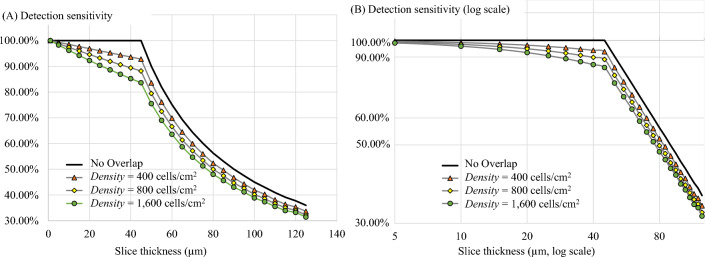
The effect of the cell overlapping on the detection sensitivity. With no overlap in the cell signals (black line), the detection sensitivity starts to decrease exactly at the optimal slice thickness point. With the overlap, the sensitivity linearly decreased started from the beginning (*X* = 1 µm) until reaching the optimal slice thickness (*X* = 44 µm) where the sensitivity started to decrease hyperbolically. With higher degree of cell overlapping (as determined by *Density*), the sensitivity decreased at a higher rate. Although the optimal points are well-defined in all situations (at *X* = 44 µm), the perfect sensitivity could not be maintained in all situations (*Sens* < 100%).

Last, the cell overlapping effect was tested by randomly placing the model cells within a fixed area of the virtual tissue to allow superpositions of their sub-surface fluorescence. The degree of overlapping is governed by *Density* which is calculated by the number of model cells to tissue area. Figure 16 illustrates the cell signals and their detectable signals in three virtual tissues, each with different *Density* values. We denoted some of the overlapping points in Fig. 16 using yellow arrowheads. The corresponding sensitivity-thickness curves are shown in Fig. 17. As compared to the no-overlap case, the sensitivity curves in the overlapping cases linearly decreased from the beginning (*X* = 1 µm) until reaching the optimal point (at *X* = 44 µm) where the curves started to decrease hyperbolically. Interestingly, the optimal points were well-defined in all situations (*X* = 44 µm), but the sensitivities could no longer guarantee at 100% at any point (except *X* = 1 µm). With higher degrees of cell overlapping (as determined by *Density*), the sensitivity curve decreased at a higher rate.

## Discussion

We created a model suitable for guiding selection of slice thickness in section-and-image systems for applications of cell detection (e.g., stem cells and micro-metastases) and microspheres. The model explicitly describes factors that contributed to the fluorescent cell detectability in cryo-imaging for the first time. The key factors were the section thickness (*X*), the fluorescent cell intensity (*I*_*fluo*_), the effective tissue attenuation coefficient (*μ*_*T*_), and a detection threshold of the system (*T*). The model suggests the value of the optimal slice thickness [*X*_*optimal*_ in Eq. (5)] which is the largest slice thickness that guarantees 100% cell detection. This optimal slice thickness does not only enable the cryo-imaging system to acquire and detect all fluorescent cells in tissue sample, but it also allows users to expedite the imaging process as well as to extend the service life of the sectioning knife and other mechanical parts. The model was built upon the data derived from cryo-imaging technique under some key assumptions. We speculate that the model could be applicable to other block-face imaging technique such as serial block-face scanning electron microscopy (SBEM), Microscopy with UV Surface Excitation (MUSE) imaging, etc.

In this work, we presented the relationship between the slice thickness (*X*) and the detection sensitivity (*Sens*) for the first time. This relationship was established under the key assumptions stating that all intensity stable cells were evenly distributed with no overlap in a homogeneous tissue. With these assumptions, we could derive mathematical models predicting the number of cell detections Eq. (11) and the *Sens* values Eqs. (13)–(14) for each sub-optimal choice of the slice thickness (*X* > *X*_*optimal*_). After running in silico simulations based on the assumptions, we made the following observations. When *X* ≤ *X*_*optimal*_, the *Sens* value was constant at 100%_*,*_ but when *X* > *X*_*optimal*_, it decreased as a non-linear function of *X* (Fig. 6A). The relationship in the sub-optimal range was shown to be a reciprocal function of *X* Eq. (14). When plotted on a log–log scale, the relationship was linear with a slope of -1 (Fig. 6B). We speculate that this relationship would hold true for real data if the fluorescent cells behaved in a manner that was consistent with our key assumptions. Please note that we also developed a probabilistic model to predict the sensitivity profile in cases where the cell distribution is not uniform Eqs. (7)–(10) and with varying intensity Eqs. (16)–(17).

We propose that the relationship could be used to estimate the true number of cells when one chooses a slice thickness that is too large (*X* > *X*_*optimal*_). In many applications, it is necessary for cryo-imaging users to choose a larger slice thickness in order to analyze a large piece of tissues such as pig hearts^5,22,23,27^, dog hearts^7,21^, or rabbit tissues^24^. As the sample volume is prohibitively large, a much larger slice thickness should be selected in order to efficiently optimize the imaging time, the memory space, and other costs. However, the larger the slice thickness beyond the *X*_*optimal*_ value was, the higher the number of false negatives. In order to mitigate this issue, we propose that the relationship derived in Eqs. (14)–(15) can be used to estimate the true number of cells/microspheres. For example, if researchers conducted a microsphere biodistribution study in a large animal and needed to set a large slice thickness, say *X* = 100 μm while *X*_*optimal*_ = 58 μm. By doing so, the cell signals acquired in their image data could be obtained with the sensitivity of only 58% (42% lost) as suggested by Eqs. (14)–(15). For correcting this, one may consider multiplying the number of found microspheres by a factor of 1/*Sens* (1/0.58 in our example) for estimating the true number of fluorescent microspheres in the tissue. This knowledge allows cryo-imaging operators to use larger slice thickness (much larger than *X*_*optimal*_) without significant loss of the microspheres. Such corrections could enable reduced scanning time of a large tissue such as a pig heart, going from days or weeks to hours or days. This finding makes that analyses feasible that may not previously be otherwise.

We successfully validated the model using real data from two independent studies. The model describes well the observations from the real data. The first dataset was from an experiment studying fluorescently labeled stem cells in a mouse model^2,9,11,12^. The second dataset was from another experiment studying fluorescent microsphere biodistribution in a pig heart^5,26^. By performing section-and-image simulation on these datasets, the measured slice thickness and cell detection relationships were found to be well correlated with those from in silico simulation results (Figs. 10 and 11). The correlations between the curves were more than 99%. We believe that this high degree of matching was largely due to the fact that the fluorescent cell/microsphere distribution in the datasets conformed with our assumptions (Figs. 4B and 5). Currently, we are working on cases where the cell distribution in the sample is not consistent with the assumptions due to overlap or varied intensity. Examples of these are pulmonary emboli in a lung tissue after intravenous injection of the cells (Fig. 4A), a tumor mass or metastasis in a mouse model^4,13–19^, an aggregation of activated immune cells in secondary lymphoid organs^3,9,11,12^, etc.

By having a brighter fluorescent intensity, the slice thickness could be set to a larger value. Under the current cryo-imaging protocol^1,5,9,10^, the datasets show that the fluorescent intensity values (*I*_*fluo*_) of red microsphere, green microsphere, and the quantum dot labeled stem cells were about 300, 87, and 38, respectively. The estimated optimal slice thicknesses for these fluorescent microspheres and cells were 102, 58, and 43 μm, respectively. If slice thickness must be fixed, high cell brightness must be maintained so that signal presence in the output images could be guaranteed. Our model suggests the cell intensity should not be lower than the minimum cell brightness *I*_*optimal*_ as described in Eq. (6). Since the fluorescently labeled cells were much dimmer than the fluorescent microspheres, researchers need to ensure that the cells are fluorescently labeled with the intensity greater than the recommended value. This could be done by several means. One example is to select only the bright cells through the fluorescence-activated cell sorting (FACS) machine before the in vivo delivery. One should consider that cell division, cell death, photobleaching of the fluorophores, and other factors could contribute to the cell brightness variation. If a rapid loss in the cell brightness is expected, one might use a smaller slice thickness to compensate for it. Another approach to increase the cell brightness is to increase the exposure time of the camera. Because of high signal to noise ratio of the fluorophores, longer exposure time usually increases contrast of the cells of interest. Again, brighter cells allow the operators to use larger slice thickness as described in Eq. (5). However, by increasing the exposure time, it will lead to longer overall scan time. For scanning a pig heart (10 cm sample thickness with 20 µm slice thickness, 5 × 5 tiles per slice), it can take up to 3 days with a 1 s exposure time. By increasing the exposure time to 2 s, the sample would need about a week to image. One would need to optimize the exposure time and the slice thickness to balance the machine workload.

We recommend researchers to perform calibration experiments for generating “the sensitivity vs. slice thickness” relationship prior to real experiments as described in Pseudocode 2. The relationship would reveal the optimal slice thickness values as shown in Fig. 10. The calibration experiment can be performed by sectioning the test tissue with a small slice thickness. Section-and-image simulation is then applied to the data to generate the relationship between different values of slice thickness and detectability. The calibration experiments ensure that the slice thickness is optimized for different experiments where the cell brightness is varying.

Moreover, we reported the detectability characteristics of situations where the key assumptions did not hold (Figs. 12, 13, 14, 15, 16, 17). The simulations were to study the effects of (1) tissue inhomogeneity, (2) cell brightness variations, and (3) cell signal overlapping on the detectability as observed in the sensitivity-thickness curves (Figs. 13, 15, 17). Different situations resulted in different unique patterns in the sensitivity-thickness curves as explained in the Results section. We believe that these characteristics of the curves can be used to assess if the key assumptions hold in any experiment. In all simulations, the detection sensitivities were not at 100% even if the operator cut the sample with the optimal slice thickness [as determined by Eq. (5)]. The optimal points tend to retrace back further (toward smaller values) depending on the degree of the key assumption violation. Thus, we recommend the operator to use a smaller slice thickness to minimize the sensitivity reduction if the violation is expected. Oftentimes, researchers analyze one organ at a time instead of analyzing a sample of mixed different tissues. For example, cell/microsphere distribution in a single organ as presented in this paper. In such a case, the effect of the *µ*_*T*_ variability can be minimal because the parameter value can be estimated separately for each tissue type. Some problems, such as cell signal overlap or irregular distribution, can be solved digitally using image processing techniques. Our group^30,34^ as well as others^7^ proposed algorithms that can effectively separate two (or more) overlapping sub-surface fluorescent signals in the cryo-imaging data.

Finally, we showed that cryo-imaging technology could be used to track fluorescent cells and microspheres anywhere in the entire animal with microscopic sensitivity. In this study, datasets from two different experiments were used. These included fluorescently labeled cells in a mouse model (Fig. 4) and fluorescent microspheres in a pig heart (Fig. 5). With carefully chosen imaging parameters, cryo-imaging could yield a recovery rate (number of detections/delivery) as high as 94%. We believe that cryo-imaging could provide image data with similar recovery rates in other biological applications such as drug delivery, blood perfusion, cancer metastasis, etc.

In conclusion, we presented a model describing the relationship between the fluorescent cell detection and the slice thickness for a section-and-image cryo-imaging system. The model could be used to suggest the optimal slice thickness value that guarantees ideal detection of fluorescent cells while minimizing scan time. We also successfully validated the model using fluorescent microsphere data and fluorescently labeled stem cell data. The model also provides a correction factor to account for degraded sensitivity at suboptimal slice thickness. We recommend acquiring a thin slice calibration dataset for every scan that involves absolute quantification of fluorophore spatial distribution, thus serving as quality assurance in the case that a correction is needed. As cryo-imaging technology has been used in many biological applications, this work plays a role in increasing both experimental throughput and quality assurance, thus further advancing its usability and reliability.

## Supplementary Information

Supplementary Information.

## Data Availability

The datasets and the source codes, that are used/generated/analyzed during the current study, are available from the corresponding author on reasonable request.
