# Supplementary material for: Optimal slice thickness for improved accuracy of quantitative analysis of fluorescent cell and microsphere distribution in cryo-images
Source: Sci Rep. 2023 Jul 5;13:10907. doi: 10.1038/s41598-023-37927-y (PMC10322852; doi:10.1038/s41598-023-37927-y)
Supplement: Supplementary file 1 — Supplementary Information. [file 41598_2023_37927_MOESM1_ESM.docx]

Optimal Slice Thickness for Improved Accuracy of Quantitative
Analysis of Fluorescent Cell and Microsphere Distribution in
Cryo-images: Supplemental Data

Patiwet Wuttisarnwattana^a,*^, Brendan L. Eck^b, d^, Madhusudhana Gargesha^c^, and
David L. Wilson^d,*^

^a^ Biomedical Engineering Institute, Department of Computer Engineering, Excellence Center in Infrastructure Technology and Transportation Engineering, Chiang Mai University, Chiang Mai, 50200, Thailand;

^b^ Imaging Institute, Cleveland Clinic, Cleveland, OH 44195, USA;

^c^ BioInVision Inc., Mayfield Village, OH 44143, USA;

^d^ Department of Biomedical Engineering, Case Western Reserve University, Cleveland, OH, 44106, USA

**Examples of the fluorescent images used in our study**

To test the validity of our model, we compared the simulated results to real data. There were two datasets that we used in this study: (1) fluorescently labeled stem cells in a mouse tissue [^1-3^](#_ENREF_1), and (2) fluorescent microspheres in a pig model [^4^](#_ENREF_4)^,^[^5^](#_ENREF_5). In the first dataset, image data consisted of a mouse lung containing fluorescently labeled stem cells. In this experiment, the lung was removed from a mouse. It was directly injected with 1 × 10^5^ fluorescent stem cells. Fluorescent and brightfield images of the whole sample were acquired via cryo-imaging. A raw image zooming to the fluorescent cell signals is shown in Suppl. Fig. 1A. The images were then stacked, registered, and visualized in 3D in Fig. 2B (in the main article). In the second dataset, we used images of a pig heart, containing fluorescent microspheres, to perform the simulation. Examples of the fluorescent microsphere signals and the 3D visualization are shown in Suppl. Fig. 1B and Fig. 3 (in the main article), respectively.


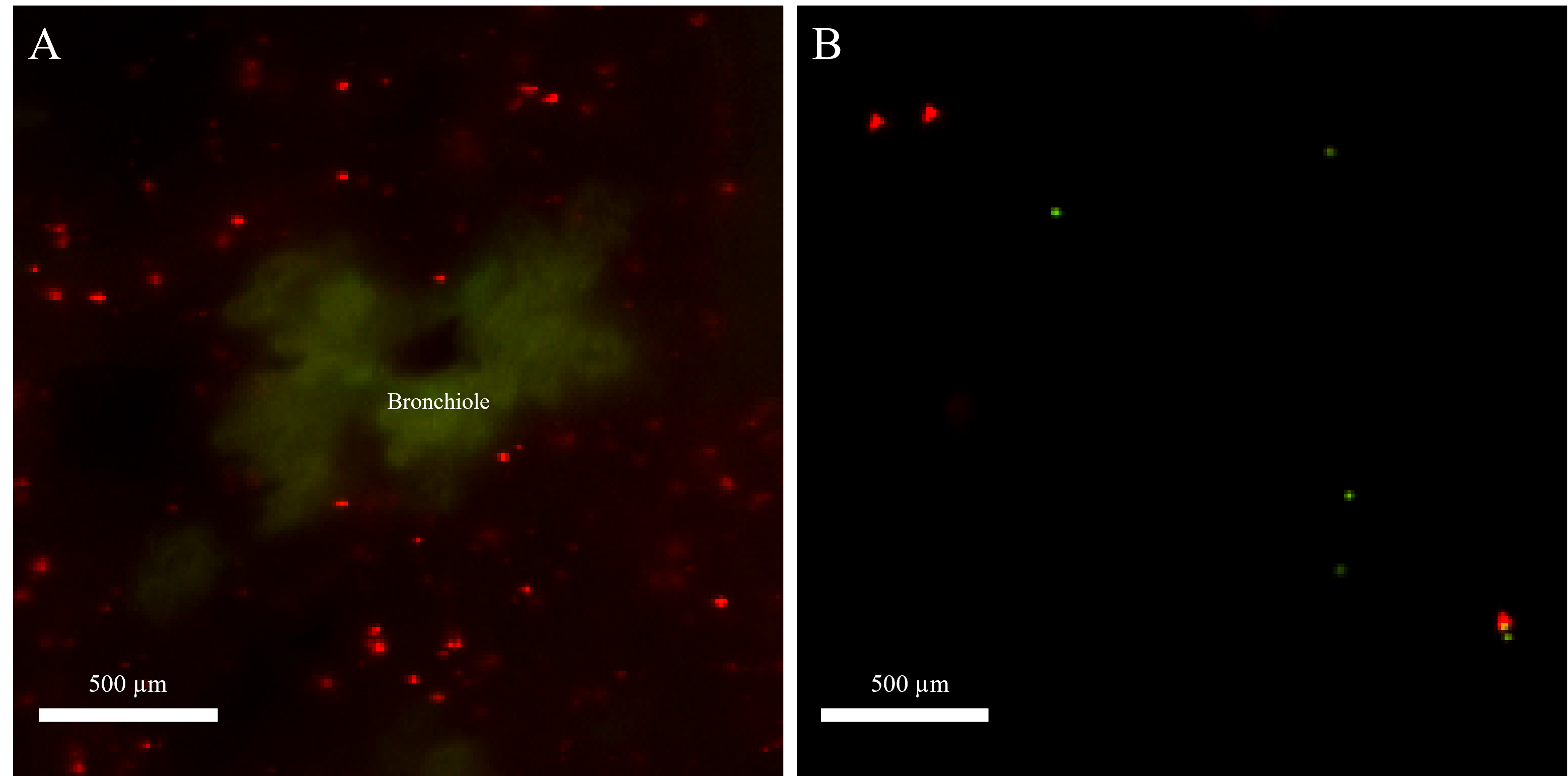


Suppl. Fig. 1. Two sections, each from different experiments, showing examples of fluorescent signals of stem cells and microspheres. (A) stem cells were labeled with red quantum dots and directly injected into a mouse’s lung. Figure A shows the stem cell signals (red) in the tissue. Autofluorescence of a bronchiole can be seen in the middle. (B) In another experiment, fluorescent microspheres were used to measure myocardial blood perfusion in a pig model. Figure B shows fluorescent signals of red and green microspheres entrapped in a pig myocardium. Notice that there was no or less autofluorescent signals in the heart tissue (B) as compared to the lung tissue (A).

**Animal Experiments**

We validated our model using animal datasets from previous studies. We employed cryo-imaging data from two experiments: fluorescent microspheres in porcine tissue [^4^](#_ENREF_4)^,^[^5^](#_ENREF_5) and fluorescently labeled stem cells in a whole mouse [^1^](#_ENREF_1)^,^[^2^](#_ENREF_2)^,^[^6^](#_ENREF_6). Readers are encouraged to review the provided citations for details about the experimental protocols and the original research. In this study, we used these secondary data for validating our model. All experiments were approved by the Institutional Animal Care and Use Committee (IACUC) at Case Western Reserve University. In the following paragraphs, we briefly describe the experiments.

*1. Microspheres in a porcine ischemia model*

For the microsphere experiment, a porcine model of ischemia was used. The original paper aimed to compare myocardial blood flow (MBF) measurements determined using CT imaging versus the fluorescent microsphere deposit method [^4^](#_ENREF_4)^,^[^5^](#_ENREF_5). Pigs (Yorkshire female, weight 40-50 kg, age 13-15 weeks) were percutaneously induced with ischemia guided by the fractional flow reserve (FFR) protocol [^4^](#_ENREF_4)^,^[^5^](#_ENREF_5)^,^[^7^](#_ENREF_7). Under fluoroscopy, a pigtail catheter for fluorescent microsphere injection was introduced in the femoral artery and placed in the left ventricle cavity. The balloon was inflated to induce the desired level of stenosis as determined by FFR. Two colors of fluorescent microspheres were separately injected into the animals during the ischemic and baseline conditions. During the baseline condition (FFR=1.0), 9 million red microspheres (15 µm, 625 nm excitation, 645 nm emission, Molecular Probes Inc.) were injected, followed by a saline flush. During the ischemic condition (FFR=0.7), 9 million green microspheres (15 µm, excitation 495 nm, 525 nm emission, Triton Technology Inc.) were injected. The microsphere injections were performed in the animal surgery suite followed by CT imaging for further analysis. After CT scanning, the animals were sacrificed, and the hearts were excised. The samples were embedded in “Optimal Cutting Temperature” compound (OCT, Tissue Tek) and flash frozen with liquid nitrogen. Cryo-imaging of the sample was performed using the CryoViz cryo-imaging system (BioInVision Inc., OH). Brightfield and fluorescent images were acquired with 10.7 µm in-plane resolution and 10 µm slice thickness. Since the whole heart dataset was very large, we used only a small part of an apex section of the heart tissue in our study. Approximately 5 thousand microspheres were detected in this dataset. Examples of the fluorescence data are shown in Suppl. Fig. 1B and Fig. 4 in the main article.

*2. Stem cells in a murine graft-versus-host disease model*

For the stem cell experiment, a murine graft-versus-host disease (GVHD) model was used. The original work aimed to track biodistribution of stem cells in the mouse model and investigate their immunomodulation effects. Two types of stem cells were studied: mesenchymal stem cells (MSCs) and multipotent adult progenitor cells (MAPCs). GVHD was induced in mice using allogeneic bone marrow transplantation [^2^](#_ENREF_2)^,^[^6^](#_ENREF_6). Female C57BL/6J (B6) donor and B6D2F1 (F1) recipient mice (aged 8-12 weeks) were purchased from Jackson Laboratory (Bar Harbor, ME). Total body irradiation was given to the recipient mice to eliminate the host bone marrow and their immune cells. Bone marrow cells and immune cells, extracted from B6 donors, were intravenously injected into F1 recipients to reinstitute hematopoietic cells as well as to induce GVHD symptoms in the host. MSCs and MAPCs are widely known for their GVHD alleviation effects in the mouse model. The stem cells were fluorescently labeled with red quantum dots (Qtracker 625, Thermo Fisher Scientific) and intravenously injected to the mice. The cells were fluorescence-activated cell sorted (FACS) before delivery to ensure a high cell brightness. Animals were euthanized and prepared for cryo-imaging. A specialized stem cell detection algorithm was developed to detect, quantify, and visualize cells in the whole mouse cryo-imaging data [^8^](#_ENREF_8). Stem cell biodistribution in different tissues have been identified and reported in previous studies [^1^](#_ENREF_1)^,^[^2^](#_ENREF_2)^,^[^6^](#_ENREF_6). In this study, we used two mouse datasets - the first was a mouse injected with 5 × 10^5^ red fluorescently labeled MAPCs. In this experiment, the slice thickness was set to 40 μm. We visualized and quantified the number of cells detected in the mouse. In the second mouse, MSCs were used instead of MAPCs. About 1 × 10^5^ MSCs were fluorescently labeled with red quantum dots (Qtracker 605) and then sorted using FACS to ensure a high cell brightness. The cells were directly injected into an excised mouse lung and the lung was subsequently cryo-imaged. The slice thickness was set to 20 μm. Examples of the fluorescence data are shown in Suppl. Fig. 1A. and Fig. 3 in the main article.

Additionally, we would like to show that cryo-imaging technology could provide an accurate quantification of fluorescently labeled cells in the mouse model with a very high recovery ratio. The recovery ratio was determined as the number of detected cells in the whole mouse data divided by the number of cells injected into the mouse. In this experiment, we had two mice (the same as above): the mouse that was intravenously injected with 5 × 10^5^ labeled cells and the mouse that was directly injected with 1 × 10^5^ labeled cells into its lung. In Fig. 3 (in the main article), we performed 3D renderings of these mice and the cells. The numbers of recovery ratios are reported in the Results section.

**References**

1 Wuttisarnwattana, P., Eid, S., Gargesha, M., Cooke, K. R. & Wilson, D. L. Cryo-imaging of Stem Cell Biodistribution in Mouse Model of Graft-Versus-Host-Disease. *Annals of biomedical engineering* (2020). <https://doi.org:10.1007/s10439-020-02487-z>

2 Auletta, J. J. *et al.* Human mesenchymal stromal cells attenuate graft-versus-host disease and maintain graft-versus-leukemia activity following experimental allogeneic bone marrow transplantation. *Stem cells* **33**, 601-614 (2015). <https://doi.org:10.1002/stem.1867>

3 Metheny, L. *et al.* Human multipotent adult progenitor cells effectively reduce graft-vs-host disease while preserving graft-vs-leukemia activity following experimental, allogeneic bone marrow transplantation. *Stem cells* **2021**, 1-14 (2021). <https://doi.org:https://doi.org/10.1002/stem.3434>

4 Eck, B. L. *et al.* Comparison of quantitative myocardial perfusion imaging CT to fluorescent microsphere-based flow from high-resolution cryo-images. *Proceedings of SPIE--the International Society for Optical Engineering* **9788** (2016). <https://doi.org:10.1117/12.2217027>

5 Fahmi, R. *et al.* Quantitative myocardial perfusion imaging in a porcine ischemia model using a prototype spectral detector CT system. *Phys Med Biol* **61**, 2407-2431 (2016). <https://doi.org:10.1088/0031-9155/61/6/2407>

6 Metheny, L. *et al.* Human multipotent adult progenitor cells effectively reduce graft-vs-host disease while preserving graft-vs-leukemia activity following experimental, allogeneic bone marrow transplantation. *Stem cells* **n/a** <https://doi.org:https://doi.org/10.1002/stem.3434>

7 De Bruyne, B. & Sarma, J. Fractional flow reserve: a review: invasive imaging. *Heart* **94**, 949-959 (2008). <https://doi.org:10.1136/hrt.2007.122838>

8 Wuttisarnwattana, P., Gargesha, M., van't Hof, W., Cooke, K. R. & Wilson, D. L. Automatic Stem Cell Detection in Microscopic Whole Mouse Cryo-Imaging. *IEEE Trans Med Imaging* **35**, 819-829 (2016). <https://doi.org:10.1109/TMI.2015.2497285>
